# Hierarchical topological model of the factors influencing adolescents' non-suicidal self-injury behavior based on the DEMATEL-TAISM method

**DOI:** 10.1038/s41598-022-21377-z

**Published:** 2022-10-14

**Authors:** Zhensong Lan, Kee Pau, Hapsah Mohd Yusof, Xuefang Huang

**Affiliations:** 1 School of Public Administrations, Hechi University, Hechi, 546300 China; 2 Department of Psychology and Counseling, Faculty of Human Development, Sultan Idris Education University, 35900 Tanjong Malim, Perak Malaysia

**Keywords:** Psychology, Human behaviour

## Abstract

This study analyzed the factors influencing adolescents' non-suicidal self-injury (NSSI) behavior and their interrelationships, and established a total influencing factor model. Through a literature analysis and semi-structured interviews with 87 adolescents and 27 experts in Guangxi Province, China, 13 influencing factors were identified from six aspects: physiological factors, cognitive factors, emotional factors, social support factors, social environment, and NSSI behavior. A system of factors influencing adolescents' NSSI behavior and a model of the factors influencing the comprehensive antagonism multilevel explanatory structure were obtained using a combination of Decision-Making Trial and Evaluation Laboratory technique and Total Adversarial Interpretive Structural Model. In descending order, NSSI, emotional state, self-efficacy, and self-cognition influenced adolescents' NSSI behavior. Social environment, exercise, and recreation had a greater impact on other factors, while NSSI, emotional regulation ability, and emotional state were greatly affected by other factors. Emotion regulation ability, self-cognition, self-efficacy, family support, school support, social support, and sleep were found to be the core factors influencing NSSI behavior of adolescents. These factors could be used to directly intervene in adolescent NSSI behavior. Timely treatment of adolescents' negative emotional states is directly conducive to preventing NSSI behavior and alleviating its severity.

## Introduction

The psychological and behavioral problems of adolescents, especially certain behaviors linked to health risks, are currently attracting considerable attention. Self-injury in adolescents is a major public health problem. Global data show that adolescent non-suicidal self-injury (NSSI) behavior not only endangers the physical and mental health of adolescents but also leads to adolescent suicide^1–4^. Studies have shown that people with NSSI behavior are at high risk of suicide, and their suicide mortality rate is 42 times higher than that of the general population^5^. In the global data, the aggregate lifetime and 12-month prevalence of non-suicidal self-injury were 22.1% (95% CI 16.9–28.4%) and 19.5% (95% CI 13.3–27.6%), respectively^6^. In China, the behavioral problems of NSSI in adolescents are extremely serious, with a prevalence of 5.4–57.4%^7–11^.

Many factors affect adolescents’ NSSI behavior, including biology, cognition, emotion, negative life events, and stress^12–19^. First, endogenous opioid peptides and other hormones affect individual perceptions of pain before affecting emotional regulation, while emotional disorders may bring NSSI behavior ^12,20–23^. It is also believed that borderline personality disorder is related to behavioral and affective disorders, so it may be an important factor affecting NSSI behavior^24,25^. The level of physical exercise undertaken may affect hormonal balance in individuals, which subsequently affects their emotional state^16,26^. So, we can explore the relationship between the role of physical exercise in regulating emotional status and NSSI behavior. Second, self-efficacy may have an impact on individual emotion regulation, and the positive and negative functions of self-efficacy can be expanded to study the impact of individual NSSI behaviors^12,27^. Furthermore, stress conditions and stress regulation outcomes may affect individual NSSI behavior^15^. Finally, peer, family, school, and other social support systems have a positive effect on the regulation of adolescent stress, while sleep quality is also related to individual stress^28–31^.

Although previous scholars have discussed the reasons for NSSI behavior, few have explained and intervened in NSSI behavior using multidimensional influencing factors. Chinese adolescents are experiencing more and more pressures from their studies and interpersonal relationships, which cannot be ignored^32,33^. Therefore, the key factors influencing adolescents' NSSI behavior must be identified to determine the main content of adolescents' NSSI behavior crisis intervention model, which is urgently required to enable improved intervention in Chinese adolescents’ NSSI behavior.

### Theoretical basis

This study is based on the multi-factor integration model^34^, the Nock integration mode^35^, and other related theories, from which the theoretical framework of the NSSI crisis intervention model was constructed. The reasons for the occurrence of NSSI lie in the management and control of negative emotions or in the way NSSI vents negative energy and alleviates the distress of negative emotions^12,36^. Cognitive theory holds that psychological disorders originate from our misunderstanding of environmental events, which directly affects our emotions, behavior and physiological state^37,38^. Multi-factor integration theory holds that NSSI behavior is caused by the interaction of biological heredity, psychological state, environmental factors, and social factors^34^. Nock's integration model emphasizes the regulation of personal emotional/cognitive experiences to promote communication with or influence others, and thus achieve emotional regulation and other functions^39^. In interventions related to NSSI behavior, social support theory holds that the social support system experienced by individuals can also play a major role in their NSSI behavior^40^. A strong social support system can alleviate an individual's negative emotional experiences, release negative energy in the heart, and reduce the occurrence of NSSI behavior^41^.

Over the years, scholars have widely explored interventions in NSSI behavior and have established a variety of explanatory models^32,42,43^. These studies provide valuable guiding theoretical and intervention methods for the clinical work of NSSI intervention. For example, drug therapy^15,44–46^ and behavioral therapy may be applied to alleviate self-punishment^32,47–49^^.^ In addition, a meta-analysis showed that dCBT-I treatment had a positive intervention effect on insomnia, which also has a positive implication for the future adoption of dCBT-I intervention in adolescents' NSSI behavior^50^. However, an integrated intervention approach might be more effective^51,52^. Therefore, this study focused on exploring the appropriate guidance for and control of adolescents' emotional and psychological reactions to help them correct cognitive, emotional, and behavioral distortions. Meanwhile, the integration of internal and external resources such as peer groups, family support, and school support would help these individuals enhance their sense of security, support, and self-efficacy as well as their ability to solve and cope with crises.

Based on a literature analysis and semi-structured interviews with adolescents and experts in related fields, the factors affecting Chinese adolescents' NSSI behavior are identified and summarized in this study, while the Decision-Making Trial and Evaluation Laboratory (DEMATEL) technique and Total Adversarial Interpretive Structural Model (TAISM) are introduced. This study aimed to explore the influencing factor model of Chinese adolescents' NSSI behavior, determine the key influencing factors, analyze the relationship between the influencing factors, and construct a preliminary NSSI behavioral crisis intervention to provide a theoretical basis for the future development of crisis intervention strategies suitable for addressing NSSI behavior among Chinese adolescents in Guangxi.

### The DEMATEL-TAISM method

The DEMATEL was developed in 1971 by Gabus and Fontela to understand complex and difficult real-world problems. This systematic analysis method uses graph theory and matrix tools^53^. Its purpose is to calculate each element's degree of influence and degree of being influenced by developing a logical relationship and direct influence matrix involving the elements in the system. This enables the cause degree and centrality degree of each element to be calculated as the basis for constructing the model, from which the causal relationships among the elements and the status of each element in the system can be determined. The essence is to regard the system as a weighted directed graph.

The Interpretive Structure Model (ISM), proposed by Professor J. Warfield in 1973, is mainly used to analyze the elements of a complex system, their interdependence, and their mutual constraints^54,55^. Its basic principles are to simplify the complex system into a molecular system (factors, elements) and use a directed graph to represent the binary relationship between subsystems, through Boolean logic operations, to construct a top-down multilevel recursive overall system structure. This is presented in the simplest form as a hierarchically directed topological graph^56–58^. Huang introduced the idea of game antagonism into Game Interpretative Structural Modeling (GISM)^59^, while Murray et al. first proposed the cross-impact analysis-interpretive structural modeling method^60^. In his research, Liu used the perspective of system theory to analyze the game explanatory structural model in depth^61^, provided the process and steps of the game explanatory structural model method, improved the hierarchical division of the classical explanatory structural model, and analyzed the hierarchy obtained by the hierarchical extraction method of "result priority" or "reason priority." Thus, a more reasonable topological hierarchical graph is obtained. Ni and Huang further optimized and integrated the adversarial idea into a Generative Antagonistic Network, after which they proposed a new model method (the Adversarial Interpretive Structure Modeling Method, AISM)^62^.

To illustrate more clearly the relationships between the subsystems in the topological hierarchy, values were assigned to the correlation and a TAISM was proposed. This was used to construct a model of the factors influencing NSSI behavior among adolescents in Guangxi, China.

## Results

### A comprehensive matrix of influencing factors of adolescents' NSSI behavior

The direct impact matrix, O, was obtained according to the comprehensive scores given by the expert team, as shown in Table 1. Based on the direct influence matrix O, normalization processing was performed using formula (1) to obtain the normalization matrix N, as shown in Table 2. Then, the calculation and conversion were performed using formula (2) to obtain the comprehensive influence matrix T, as shown in Table 3.

**Table 1:** Direct effects on the matrix O.

		A2	A3	B2	B3	B4	B5	B6	B7	B8	B9	B10	B11	Y
O =	A2	0	67	15	18	82	35	19	12	11	11	11	16	85
	A3	81	0	17	18	85	32	18	12	11	11	11	15	46
	B2	15	13	0	102	23	79	59	18	17	18	18	14	82
	B3	15	13	84	0	18	83	62	15	16	16	12	16	78
	B4	43	13	22	23	0	22	20	14	14	14	14	34	94
	B5	43	12	20	19	85	0	24	16	12	12	12	36	97
	B6	21	19	42	72	30	61	0	12	13	13	13	27	74
	B7	12	12	19	16	18	94	21	0	71	73	17	14	75
	B8	11	14	18	19	21	81	22	77	0	70	18	16	62
	B9	11	15	20	19	21	64	20	76	72	0	13	17	65
	B10	11	15	72	19	21	62	21	63	76	73	0	23	70
	B11	20	19	18	50	22	78	21	15	19	15	15	0	85
	Y	25	11	21	22	18	15	10	15	12	12	11	10	0

“A2, ” “A3,” “B2,” “B3,” “B4,” “B5,” “B6,” “B7,” “B8,” “B9,” “B10,” “B11,” and “Y” stand for "Sleep,” "Exercise,” "Self-cognition,” "Self-efficacy,” "Emotional state,” "Emotion Regulation Ability,” “Peer Support,” "Family Support,” "School Support,” "Social Support,” "Social Environment,” "Leisure Entertainment,” and “NSSI”.

**Table 2:** Normalization matrix N.

		A2	A3	B2	B3	B4	B5	B6	B7	B8	B9	B10	B11	Y
N =	A2	0	0.072	0.016	0.019	0.088	0.038	0.02	0.013	0.012	0.012	0.012	0.017	0.091
	A3	0.087	0	0.018	0.019	0.091	0.034	0.019	0.013	0.012	0.012	0.012	0.016	0.049
	B2	0.016	0.014	0	0.11	0.025	0.085	0.063	0.019	0.018	0.019	0.019	0.015	0.088
	B3	0.016	0.014	0.09	0	0.019	0.089	0.067	0.016	0.017	0.017	0.013	0.017	0.084
	B4	0.046	0.014	0.024	0.025	0	0.024	0.021	0.015	0.015	0.015	0.015	0.037	0.101
	B5	0.046	0.013	0.021	0.02	0.091	0	0.026	0.017	0.013	0.013	0.013	0.039	0.104
	B6	0.023	0.02	0.045	0.077	0.032	0.066	0	0.013	0.014	0.014	0.014	0.029	0.079
	B7	0.013	0.013	0.02	0.017	0.019	0.101	0.023	0	0.076	0.078	0.018	0.015	0.081
	B8	0.012	0.015	0.019	0.02	0.023	0.087	0.024	0.083	0	0.075	0.019	0.017	0.067
	B9	0.012	0.016	0.021	0.02	0.023	0.069	0.021	0.082	0.077	0	0.014	0.018	0.07
	B10	0.012	0.016	0.077	0.02	0.023	0.067	0.023	0.068	0.082	0.078	0	0.025	0.075
	B11	0.021	0.02	0.019	0.054	0.024	0.084	0.023	0.016	0.02	0.016	0.016	0	0.091
	Y	0.027	0.012	0.023	0.024	0.019	0.016	0.011	0.016	0.013	0.013	0.012	0.011	0

“A2, ” “A3,” “B2,” “B3,” “B4,” “B5,” “B6,” “B7,” “B8,” “B9,” “B10,” “B11,” and “Y” stand for "Sleep,” "Exercise,” "Self-cognition,” "Self-efficacy,” "Emotional state,” "Emotion Regulation Ability,” “Peer Support,” "Family Support,” "School Support,” "Social Support,” "Social Environment,” "Leisure Entertainment,” and “NSSI”.

**Table 3:** Synthesis influence matrix T.

		A2	A3	B2	B3	B4	B5	B6	B7	B8	B9	B10	B11	Y
T =	A2	0.023	0.081	0.034	0.039	0.112	0.065	0.036	0.027	0.025	0.025	0.021	0.031	0.135
	A3	0.103	0.015	0.035	0.038	0.115	0.062	0.034	0.026	0.025	0.025	0.02	0.03	0.096
	B2	0.038	0.028	0.029	0.132	0.055	0.125	0.085	0.038	0.036	0.037	0.03	0.032	0.145
	B3	0.037	0.027	0.109	0.031	0.049	0.125	0.086	0.033	0.034	0.033	0.024	0.033	0.138
	B4	0.06	0.025	0.04	0.043	0.021	0.052	0.035	0.028	0.028	0.028	0.023	0.047	0.137
	B5	0.064	0.026	0.04	0.041	0.112	0.031	0.041	0.032	0.027	0.027	0.022	0.052	0.149
	B6	0.042	0.032	0.067	0.099	0.058	0.1	0.021	0.029	0.029	0.029	0.024	0.043	0.129
	B7	0.033	0.026	0.041	0.04	0.049	0.139	0.041	0.026	0.096	0.098	0.029	0.032	0.135
	B8	0.032	0.028	0.04	0.043	0.051	0.126	0.042	0.103	0.025	0.095	0.03	0.033	0.121
	B9	0.031	0.028	0.041	0.042	0.049	0.108	0.04	0.101	0.096	0.024	0.025	0.033	0.122
	B10	0.035	0.031	0.101	0.052	0.055	0.12	0.048	0.096	0.107	0.104	0.014	0.043	0.142
	B11	0.04	0.032	0.04	0.072	0.05	0.113	0.04	0.032	0.035	0.031	0.025	0.014	0.136
	Y	0.035	0.019	0.033	0.035	0.032	0.035	0.02	0.025	0.022	0.021	0.017	0.018	0.026

“A2, ” “A3,” “B2,” “B3,” “B4,” “B5,” “B6,” “B7,” “B8,” “B9,” “B10,” “B11,” and “Y” stand for "Sleep,” "Exercise,” "Self-cognition,” "Self-efficacy,” "Emotional state,” "Emotion Regulation Ability,” “Peer Support,” "Family Support,” "School Support,” "Social Support,” "Social Environment,” "Leisure Entertainment,” and “NSSI”.

### Total adversarial interpretative structural model of influencing factors of NSSI behavior among adolescents

Based on the comprehensive influence matrix T, adjacency matrix A was obtained using formulas (3–5), as shown in Table 4. The reachability matrix R was calculated using formulas (6–7), as shown in Table 5. The general skeleton matrix S′ was obtained by further processing using Eq. (8), and S was obtained by conversion (Table 6).

**Table 4:** Adjacency matrix A.

		A2	A3	B2	B3	B4	B5	B6	B7	B8	B9	B10	B11	Y
A =	A2	0	0	0	0	1	0	0	0	0	0	0	0	1
	A3	1	0	0	0	1	0	0	0	0	0	0	0	1
	B2	0	0	0	1	0	1	0	0	0	0	0	0	1
	B3	0	0	1	0	0	1	0	0	0	0	0	0	1
	B4	0	0	0	0	0	0	0	0	0	0	0	0	1
	B5	0	0	0	0	1	0	0	0	0	0	0	0	1
	B6	0	0	0	1	0	1	0	0	0	0	0	0	1
	B7	0	0	0	0	0	1	0	0	1	1	0	0	1
	B8	0	0	0	0	0	1	0	1	0	1	0	0	1
	B9	0	0	0	0	0	1	0	1	1	0	0	0	1
	B10	0	0	1	0	0	1	0	1	1	1	0	0	1
	B11	0	0	0	0	0	1	0	0	0	0	0	0	1
	Y	0	0	0	0	0	0	0	0	0	0	0	0	0

“A2, ” “A3,” “B2,” “B3,” “B4,” “B5,” “B6,” “B7,” “B8,” “B9,” “B10,” “B11,” and “Y” stand for "Sleep,” "Exercise,” "Self-cognition,” "Self-efficacy,” "Emotional state,” "Emotion Regulation Ability,” “Peer Support,” "Family Support,” "School Support,” "Social Support,” "Social Environment,” "Leisure Entertainment,” and “NSSI”.

**Table 5:** Reachability matrix R.

		A2	A3	B2	B3	B4	B5	B6	B7	B8	B9	B10	B11	Y
R =	A2	1				1								1
	A3	1	1			1								1
	B2			1	1	1	1							1
	B3			1	1	1	1							1
	B4					1								1
	B5					1	1							1
	B6			1	1	1	1	1						1
	B7					1	1		1	1	1			1
	B8					1	1		1	1	1			1
	B9					1	1		1	1	1			1
	B10			1	1	1	1		1	1	1	1		1
	B11					1	1						1	1
	Y													1

“A2, ” “A3,” “B2,” “B3,” “B4,” “B5,” “B6,” “B7,” “B8,” “B9,” “B10,” “B11,” and “Y” stand for "Sleep,” "Exercise,” "Self-cognition,” "Self-efficacy,” "Emotional state,” "Emotion Regulation Ability,” “Peer Support,” "Family Support,” "School Support,” "Social Support,” "Social Environment,” "Leisure Entertainment,” and “NSSI”.

**Table 6:** General skeleton matrix S.

		A2	A3	B2	B3	B4	B5	B6	B7	B8	B9	B10	B11	Y
S =	A2					1								
	A3	1												
	B2				1		1							
	B3			1										
	B4													1
	B5					1								
	B6				1									
	B7						1			1				
	B8										1			
	B9								1					
	B10			1					1					
	B11						1							
	Y													

“A2, ” “A3,” “B2,” “B3,” “B4,” “B5,” “B6,” “B7,” “B8,” “B9,” “B10,” “B11,” and “Y” stand for "Sleep,” "Exercise,” "Self-cognition,” "Self-efficacy,” "Emotional state,” "Emotion Regulation Ability,” “Peer Support,” "Family Support,” "School Support,” "Social Support,” "Social Environment,” "Leisure Entertainment,” and “NSSI”.

**Figure 1:**
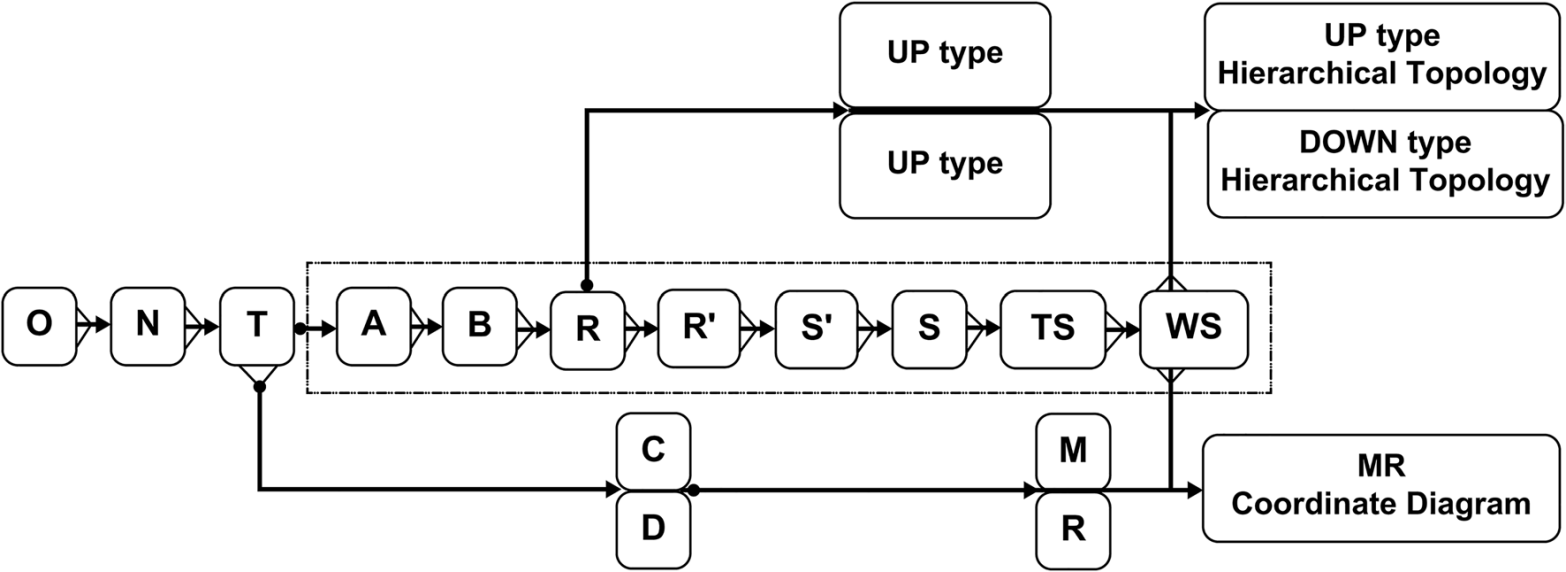
Model framework.

The degree of influence, centrality, and cause degree of each influencing factor in each model were calculated using formulas (9–12). Then, as shown in Fig. 1, the cause-result scatter diagram was drawn based on the general skeleton matrix S, with the centrality as the abscissa and the cause degree as the ordinate. The calculation results of the degree of influence, centrality, and cause degree are shown in Table 7.

**Table 7:** Impact level and Center level.

	Di	Ci	Mi	Ri
A2	0.653	0.571	1.224	0.083
A3	0.624	0.397	1.021	0.228
B2	0.811	0.65	1.461	0.16
B3	0.759	0.707	1.466	0.052
B4	0.566	0.807	1.373	− 0.242
B5	0.663	1.202	1.865	− 0.539
B6	0.701	0.57	1.271	0.131
B7	0.786	0.596	1.382	0.189
B8	0.769	0.585	1.354	0.184
B9	0.74	0.577	1.317	0.163
B10	0.949	0.302	1.252	0.647
B11	0.659	0.441	1.1	0.219
Y	0.336	1.611	1.947	− 1.275

“A2, ” “A3,” “B2,” “B3,” “B4,” “B5,” “B6,” “B7,” “B8,” “B9,” “B10,” “B11,” and “Y” stand for "Sleep,” "Exercise,” "Self-cognition,” "Self-efficacy,” "Emotional state,” "Emotion Regulation Ability,” “Peer Support,” "Family Support,” "School Support,” "Social Support,” "Social Environment,” "Leisure Entertainment,” and “NSSI”.

**Figure 2:**
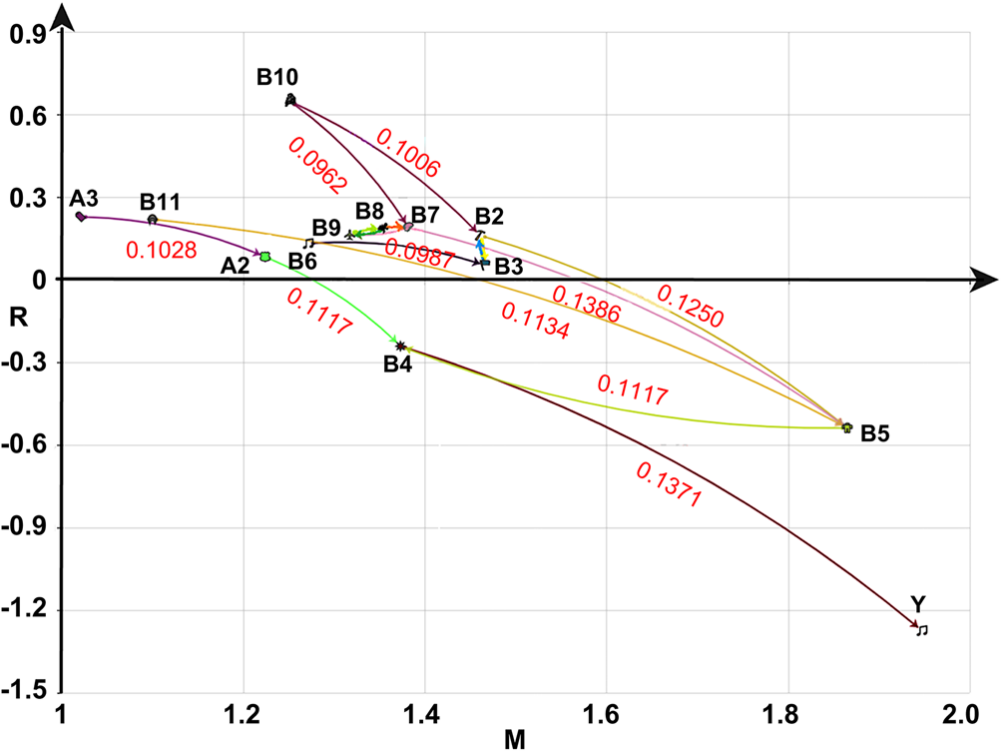
Scatter diagram of centrality and causality. “A2,” “A3,” “B2,” “B3,” “B4,” “B5,” “B6,” “B7,” “B8,” “B9,” “B10,” “B11,” and "Y" stand for "Sleep,” "Exercise,” "Self-cognition,” "Self-efficacy,” "Emotional state,” "Emotion Regulation Ability,” “Peer Support,” "Family Support,” "School Support,” "Social Support,” "Social Environment,” "Leisure Entertainment,” and “NSSI”.

The scatter plot is shown in Fig. 2. Antagonistic hierarchical division was performed according to the hierarchical extraction method, and the final hierarchical division results are listed in Table 8.

**Table 8:** Extraction results of confrontation level.

Level	Result priority—UP	Reason priority—DOWN
Level 0	Y	Y
Level 1	B4	B4
Level 2	A2, B5	B5
Level 3	A3, B2, B3, B7, B8, B9, B11	A2, B2, B3, B7, B8, B9
Level 4	B6.B10	A3, B6, B10, B11

“A2, ” “A3,” “B2,” “B3,” “B4,” “B5,” “B6,” “B7,” “B8,” “B9,” “B10,” “B11,” and “Y” stand for "Sleep,” "Exercise,” "Self-cognition,” "Self-efficacy,” "Emotional state,” "Emotion Regulation Ability,” “Peer Support,” "Family Support,” "School Support,” "Social Support,” "Social Environment,” "Leisure Entertainment,” and “NSSI”.

**Figure 3:**
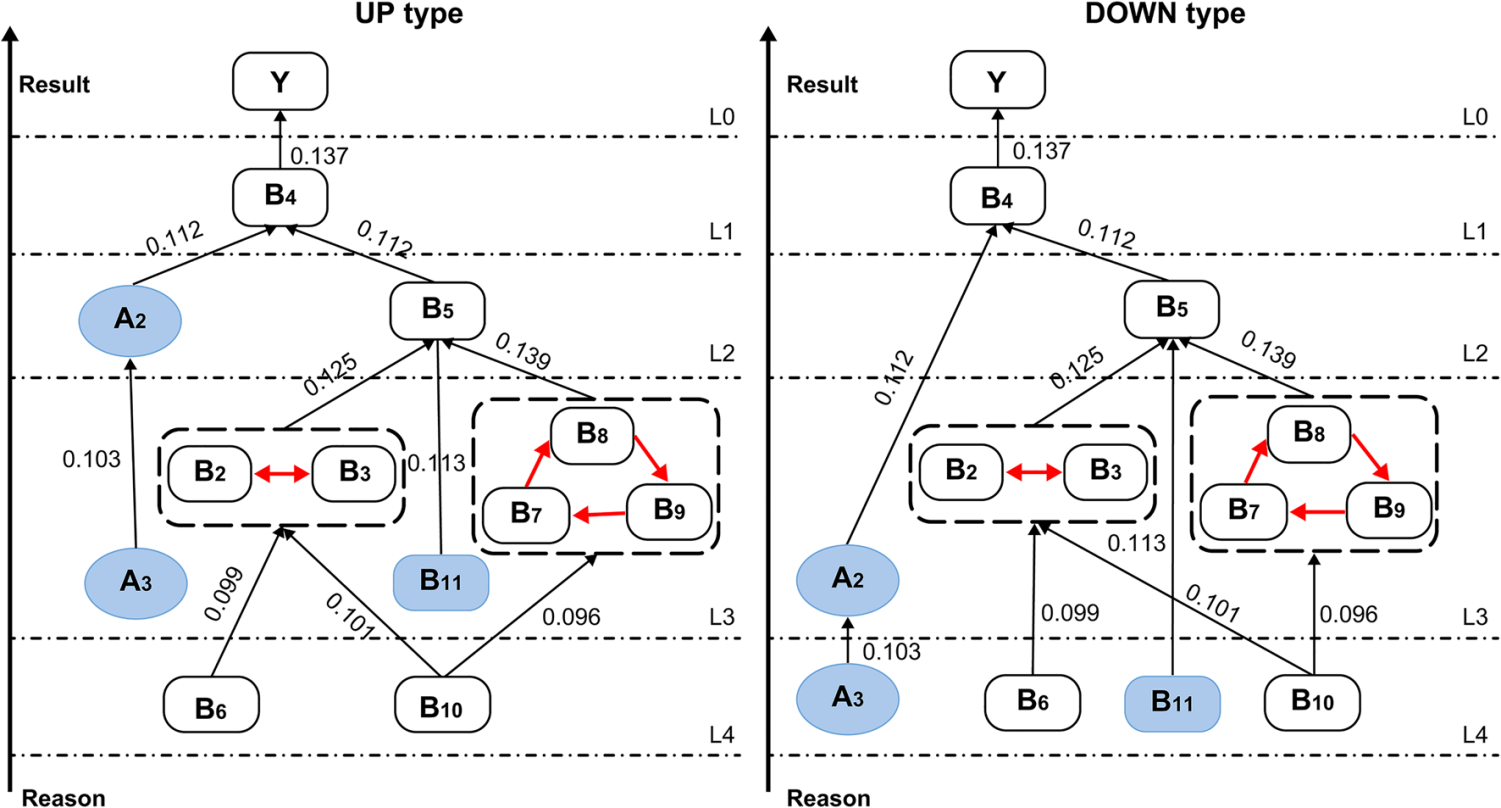
Multilevel hierarchical structure model diagram with adversarial influence coefficient. “A2,” “A3,” “B2,” “B3,” “B4,” “B5,” “B6,” “B7,” “B8,” “B9,” “B10,” “B11,” and "Y" stand for "Sleep,” "Exercise,” "Self-cognition,” "Self-efficacy,” "Emotional state,” "Emotion Regulation Ability,” “Peer Support,” "Family Support,” "School Support,” "Social Support,” "Social Environment,” "Leisure Entertainment,” and “NSSI”.

Furthermore, the hierarchical analysis results and the general skeleton matrix S enabled the creation of a multilevel hierarchical structure model of the factors influencing Chinese adolescents' NSSI behavior with an antagonistic comprehensive influence coefficient. First, the TS matrix was obtained by substituting the influence values in the comprehensive influence matrix T into the general skeleton matrix S, as shown in Table 9. The WS matrix was then obtained by replacing the values with a loop relationship with “1,” as shown in Table 10. Finally, an antagonistic topology hierarchy diagram with comprehensive influence values was obtained by substituting the response values into the antagonistic topology hierarchy diagram, as shown in Fig. 3.

**Table 9:** Matrix TS.

		A2	A3	B2	B3	B4	B5	B6	B7	B8	B9	B10	B11	Y
TS =	A2	0	0	0	0	0.1117	0	0	0	0	0	0	0	0
	A3	0.1028	0	0	0	0	0	0	0	0	0	0	0	0
	B2	0	0	0	0.1325	0	0.125	0	0	0	0	0	0	0
	B3	0	0	0.1095	0	0	0	0	0	0	0	0	0	0
	B4	0	0	0	0	0	0	0	0	0	0	0	0	0.1371
	B5	0	0	0	0	0.1117	0	0	0	0	0	0	0	0
	B6	0	0	0	0.0987	0	0	0	0	0	0	0	0	0
	B7	0	0	0	0	0	0.1386	0	0	0.0958	0	0	0	0
	B8	0	0	0	0	0	0	0	0	0	0.0948	0	0	0
	B9	0	0	0	0	0	0	0	0.1011	0	0	0	0	0
	B10	0	0	0.1006	0	0	0	0	0.0962	0	0	0	0	0
	B11	0	0	0	0	0	0.1134	0	0	0	0	0	0	0
	Y	0	0	0	0	0	0	0	0	0	0	0	0	0

“A2, ” “A3,” “B2,” “B3,” “B4,” “B5,” “B6,” “B7,” “B8,” “B9,” “B10,” “B11,” and “Y” stand for "Sleep,” "Exercise,” "Self-cognition,” "Self-efficacy,” "Emotional state,” "Emotion Regulation Ability,” “Peer Support,” "Family Support,” "School Support,” "Social Support,” "Social Environment,” "Leisure Entertainment,” and “NSSI”.

**Table 10:** Matrix WS.

		A2	A3	B2	B3	B4	B5	B6	B7	B8	B9	B10	B11	Y
WS =	A2	0	0	0	0	0.1117	0	0	0	0	0	0	0	0
	A3	0.1028	0	0	0	0	0	0	0	0	0	0	0	0
	B2	0	0	0	1	0	0.125	0	0	0	0	0	0	0
	B3	0	0	1	0	0	0	0	0	0	0	0	0	0
	B4	0	0	0	0	0	0	0	0	0	0	0	0	0.1371
	B5	0	0	0	0	0.1117	0	0	0	0	0	0	0	0
	B6	0	0	0	0.0987	0	0	0	0	0	0	0	0	0
	B7	0	0	0	0	0	0.1386	0	0	1	1	0	0	0
	B8	0	0	0	0	0	0	0	1	0	1	0	0	0
	B9	0	0	0	0	0	0	0	1	1	0	0	0	0
	B10	0	0	0.1006	0	0	0	0	0.0962	0	0	0	0	0
	B11	0	0	0	0	0	0.1134	0	0	0	0	0	0	0
	Y	0	0	0	0	0	0	0	0	0	0	0	0	0

“A2, ” “A3,” “B2,” “B3,” “B4,” “B5,” “B6,” “B7,” “B8,” “B9,” “B10,” “B11,” and “Y” stand for "Sleep,” "Exercise,” "Self-cognition,” "Self-efficacy,” "Emotional state,” "Emotion Regulation Ability,” “Peer Support,” "Family Support,” "School Support,” "Social Support,” "Social Environment,” "Leisure Entertainment,” and “NSSI”.

## Discussion

### Causality and centrality analysis

Centrality is a measure used to analyze the centrality of system elements in a system. The larger the value, the greater the importance of the element in the system. As Table 7 and Fig. 2 illustrate in descending order, the values of NSSI (Y, *w* = 1.947), emotional state (B5, *w* = 1.865), self-efficacy (B3, *w* = 1.466), and self-cognition (B2, *w* = 1.461) were the highest, indicating the importance of these factors in influencing adolescents' NSSI behavior. The values for exercise (A3, *w* = 1.021), leisure and recreation (B11, *w* = 1.1), sleep (A2, *w* = 1.224), and social environment (B10, *w* = 1.252) were relatively small, indicating that these factors have comparatively less influence on adolescents' NSSI behavior. However, it is worth noting that this does not mean that these factors are unimportant, and the important lines here are relative to other factors.

The degree of cause is a function that represents the degree of cause and effect of an element in a system.If the value is positive, this factor has a greater impact on other factors; If the value is negative, the factor is greatly affected by other factors. As Table 7 and Fig. 2 illustrate, social environment (B10, *r* = 0.647), exercise (A3, *r* = 0.228), and recreation (B11, *r* = 0.219) were found to have a greater impact on other factors, while NSSI (Y, *r* = − 1.275), emotional regulation ability (B5, *r* = − 0.539) and emotional state (B4, *r* = − 0.242) were greatly affected by other factors.

### Active system with extensibility

In a set of antagonistic topological hierarchical diagrams, active elements exist at different levels, which can also be called topologically active systems. In contrast, if an element only exists at the same level, this is a rigid requirement and becomes a rigid topological system. According to Fig. 3, the combination of A2, A3, and B11 belongs to the active element, that is, the extension system. A2 jumps from L4 to L3 and A3 and B11 jump from L3 to L2. Practically speaking, the effects of sleep (A2), exercise (A3), and the social environment (B11) on adolescents' NSSI behavior are multidimensional and multifaceted.

### Loop analysis

In an adversarial hierarchical structure diagram, the directed lines between factors indicate a causal relationship. If there is a two-way connection between two factors, there is a relationship between them, that is, there is a loop, also known as strong connectivity. In the multilevel hierarchical structure model of the factors influencing adolescents' NSSI behavior developed in this study, there are two loops in the antagonist-level topological diagram: between self-cognition (B2) and self-efficacy (B3); and among family support (B7), school support (B8), and social support (B9). This shows that self-cognition (B2) and self-efficacy (B3) as well as family support (B7), school support (B8), and social support (B9) are mutually causal.

Inappropriate self-perception among Chinese adolescents may affect their self-efficacy and lead to corresponding behaviors. It is also adolescents who habitually make wrong internal attributions to events^63^, thus strengthening their autonomous awareness of these wrong behaviors and making it easier for them to engage in NSSI behaviors. In addition, family, school, and social support form a system, and these three factors influence each other. For adolescents in middle school, family and school are important living environments, and these adolescents are also influenced by social support. This is a reminder to closely monitor the relationship between the three factors when intervening in adolescents' NSSI behavior.

### Hierarchical analysis and causal analysis

Figure 3 illustrates that the system of factors influencing adolescent NSSI behavior in Guangxi Province, China, formed a topology structure of five levels from top to bottom. In a directed line segment, the cause element points to the result element, and its full range of causes and effects is presented in Table 11. The above points indicate that the two causal full series do not coincide exactly, which is also a characteristic of an active system. However, the basic structure is similar overall; for example, there are two loop elements: [B7, B8, B9] and [B2, B3].

**Table 11:** Full series table of causality.

UP	{B6, B10} $\succ$ {[B7, B8, B9], [B2, B3], A3, B11} $\succ$ {A2, B5} $\succ$ {B4} $\succ$ {Y}
DOWN	{A3, B6, B10, B11} $\succ$ {[B7, B8, B9], [B2, B3]} $\succ$ {B5}$\succ${B4}$\succ${Y}

“A2, ” “A3,” “B2,” “B3,” “B4,” “B5,” “B6,” “B7,” “B8,” “B9,” “B10,” “B11,” and “Y” stand for "Sleep,” "Exercise,” "Self-cognition,” "Self-efficacy,” "Emotional state,” "Emotion Regulation Ability,” “Peer Support,” "Family Support,” "School Support,” "Social Support,” "Social Environment,” "Leisure Entertainment,” and “NSSI”.

Through further induction and simplification, the five levels of the system influencing behavioral factors related to adolescent NSSI were divided into three levels. The system was classified into a root layer (L4), intermediate layer (L2, L3), and result layer (L0, L1). The root-layer factors affect other factors and are at the lowest level of the figure. The intermediate-layer factors affect the upper factors, which affect the relationship at the top and the affected relationship at the bottom. The result-layer factors impact other factors, which then affect yet other factors; they are at the highest level.

In the topology diagram, the root layer emits only directed line segments. In this system, the bottom layer elements combined to form the root layer elements, {B6, B10} ∪ {A3, B6, B10.B11} = {A3, B6, B10.B11}; that is, the factor set of the root layer included four elements: exercise (A3), peer support (B6), social support (B10), and leisure and entertainment (B11). These factors are located at the highest level of the system, are unaffected by other factors, and can directly or indirectly affect other factors within the system. Root factors are dominant in the system and have the greatest impact on the NSSI behavior of adolescents in Guangxi Province, China, which is a key point to consider. In real life, leisure and entertainment need attention because Chinese parents and teachers are more focused on adolescents' learning, think that games affect learning, and neglect to provide adolescents with sufficient leisure and entertainment, which may help adolescents adjust better to their psychological status, thus helping alleviate NSSI behavior. Therefore, exercise may be an effective intervention strategy^16^.

There are several intermediate factors in this system. This factor set spans two levels and there are seven factors in total. The middle-level factor set mainly included emotion regulation (B5), self-perception (B2), self-efficacy (B3), family support (B7), school support (B8), social support (B9), and sleep (A2). These seven factor categories were the core factors influencing the NSSI behavior of adolescents in Guangxi Province, China. Positioned in the middle of the whole system and influenced by the root layer, they play a pivotal role in connecting the former and latter. They need to be prioritized in the process of intervention and could also be regarded as factors that may be used to directly intervene in adolescent NSSI behavior. The emotional state (B5) factor had an especially strong correlation with adolescents' emotions (B4) and NSSI behavior. Timely treatment of adolescents' emotional states is directly conducive to preventing and alleviating the severity of NSSI behavior among adolescents. The main function of NSSI is to regulate negative emotional experiences^12^, and this emphasizes the important role of emotion in NSSI behavior. Individuals with repeated NSSI are more likely to use escape-focused or emotion-focused coping styles^13^. Therefore, when intervening in relation to adolescents' NSSI, we should prioritize addressing their emotional state, help them master emotional regulation skills, and improve their emotion regulation ability. It is also important to focus on factors such as self-perception^64,65^, self-efficacy^12,66^, family support^26^, school support^67^, social support^68,69^, and sleep^8^, as these factors can also affect adolescents' NSSI behavior.

Therefore, the resulting factors B4 and Y are the union of the top factors, excluding the isolated factors in the figure. That is, the outcome layer factors are emotional state (B4) and NSSI (Y). The emotional state factor is the most direct constraint on NSSI behavior (Y) among adolescents in Guangxi Province, China. It directly alleviates adolescent NSSI behavior, while other factors affect the whole system through the result factor layer. Therefore, it is crucial to help teenagers regulate their emotional state.

## Conclusion

Taking Guangxi Province of China as an example, this study identified 13 influencing factors from six aspects of adolescent NSSI behavior: physiological factors, cognitive factors, emotional factors, social support factors, social environment, and NSSI. A combined model was constructed using DEMATEL and TAISM to provide theoretical support for crisis intervention services in relation to Chinese adolescents' NSSI behavior. Compared with the traditional ISM model, the DEMATEL-TAISM model was able to produce a hierarchical diagram of reverse extraction. The comprehensive system model contains influence coefficient values and can illustrate the influence between elements more intuitively, making the system model more convincing. However, owing to the limitations of the ISM model, this study still belongs to the qualitative analysis category, and the selected influencing factors cannot fully summarize the factors influencing Chinese adolescents' NSSI behavior; thus, there are certain limitations. This study further clarifies the relationship and hierarchy between the factors influencing adolescent NSSI behavior, which has important research significance for the development of crisis intervention measures related to adolescent NSSI behavior. In addition, during expert discussion and evaluation, the centrality of the impact factors may have been influenced by the interaction between the researchers. The degree of independent judgment made by the evaluation experts may have been affected, as they were more subjective. Finally, the teachers and parents of the adolescents participated in the evaluation expert group, but they may have had limited professional experience and failed to express their opinions effectively. Therefore, in future research in this field, it is crucial to select expert team members, especially those with extensive relevant research experience.

## Methods

### Identification of factors influencing adolescents' NSSI behavior

In this study, a multidimensional method was used to construct an evaluation index system for the factors influencing NSSI behavior among Chinese adolescents.

The first step was to read and arrange relevant literature. A search was then undertaken using the Web of Science, Springer, Google Scholar, CNKI Scholar, PubMed, and other literature retrieval databases using the keywords "self-injury" and "self-harm." The influencing factors, intervention strategies, and methods of NSSI behavior were selected, and the factors influencing NSSI behavior among adolescents were discussed.

In the second step, using grounded theory^70^, 84 middle school students and 27 related persons (their parents, teachers, school social workers, and experts in the field of psychological counseling and NSSI) were interviewed, and the interview results were open coded^71^. The sample of respondents came from five cities in Guangxi Province. According to the recommendation of the local education bureau, 12 middle schools in five cities were selected using the cluster sampling method in Guangxi Province, China; 48 classes of adolescents were randomly selected according to different grades to conduct a questionnaire survey, and 420 adolescents who had NSSI behavior were selected. After obtaining informed consent from adolescents and their parents, 84 adolescents were interviewed. In addition, the parents and teachers interviewed also came from families or schools from which the adolescents did. The interviewed experts were recruited mainly through invitations. The specific operation process was as follows: (a) the initial concepts were formed by coding the interview raw materials word by word and sentence by sentence; (b) after coding sentence by sentence, the similarities and differences of the concepts were compared, and the initial concepts were eliminated if their frequency was less than two times; (c) through analysis and condensation, the main factors affecting the NSSI behavior of adolescents in Guangxi, China were extracted from the raw materials. The content and purpose of the interviews were clearly reported to the interviewees before their interviews, and informed consent was obtained from them. This study was reviewed and approved by the Human Research Ethics Committee of Sultan Idris University of Education, Malaysia.

The third step was to conduct open coding based on the results of literature analysis and field interviews. After their selection, 34 influencing factors related to adolescents' NSSI behaviors were identified. Based on the system science principle, the Delphi method was used to organize a team of experts to discuss these factors. According to a suggestion made by the expert team, two factors (sleep and exercise) were added to the original 34 influencing factors. After five rounds of discussion, 36 influencing factors were subjected to deletion and rounding, and 13 influencing factors were obtained. These factors were divided into six groups: the physiological dimension, cognitive dimension, emotional dimension, social support dimension, social environment dimension, and NSSI. The index system for the influencing factors is presented in Table 12.

**Table 12:** Index system of influencing factors.

Terms	Code	Factors	Connotation
Physiological dimension	A2	Sleep	The overall sleep quality of individuals, including adequate sleep, undisturbed sleep environment, reasonable work, rest time, and the guarantee of sleep effect
	A3	Exercise	Individual physical exercise level, including the rationality of exercise frequency, duration, and intensity
Cognitive dimension	B2	Self-cognition	Individual evaluation of self and self-worth, as well as self-respect and emotional experience based on self-evaluation
	B3	Self-efficacy	Individual attribution logic of behavior and events, as well as speculation on and judgment whether they have the ability to complete a certain behavior
Emotional dimension	B4	Emotional state	The state of an individual affected by a behavior or event, including mental states such as depression, anxiety, and stress
	B5	Emotion regulation ability	Through certain strategies and mechanisms, individuals can change their emotions during physiological activities, subjective experience, expression, and behavior to meet their subjective expectations
Social support dimension	B6	Peer support	The degree of emotional communication between individuals and peers of the same or similar age, or the degree of care, companionship, and support from peers
	B7	Family support	The degree of emotional communication between individuals and their parents; the degree of care, companionship, and support of their parents; and the conditions of support
	B8	School support	This dimension includes a safe and friendly environment for students, the degree of timely and effective communication and support from teachers, and the positive evaluation of students
	B9	Social support	Whether the individual can find a communication platform from which to communicate and share in a timely and effective manner; whether the individual can provide timely, convenient, and adequate information and resource support
Social environment dimension	B10	Social environment	Individual psychology and behavior are influenced by social culture and values, as well as by an inclusive and just social environment
	B11	Leisure entertainment	Individuals enjoy the right to and opportunity for leisure and entertainment, and they enjoy a healthy, scientific, and reasonable leisure and entertainment environment. However, this is not for the purpose of overplay
NSSI	Y	NSSI	The individual will produce ideas or behavior related to NSSI

### The process of building DEMATEL-TAISM

Based on the factors influencing adolescents' NSSI behavior, the authors used the DEMATEL method to determine the causal relationships and influence intensity between the indicators through a matrix operation. The structural levels and basic elements were classified using TAISM to obtain a group of total adversarial antagonist-directed topological hierarchical graphs illustrating the influence values. The aim was to represent the interrelationships and effects of active factors in the model. Therefore, the DEMATEL-TAISM model could be used to identify and evaluate the root cause elements in a complex system and clarify its structural hierarchy. The model framework is illustrated in Fig. 1.

Wherein O is a direct influence matrix, the diagonal of which is “0”; N is a normalized influence matrix; T is a comprehensive influence matrix; A is a relational adjacency matrix obtained by taking the intercept of T; B is a multiplication matrix; R is an accessible matrix; R′ is a contracted point accessible matrix (that is, loop elements in the accessible matrix are reduced into one element); S′ is a skeleton matrix, which is obtained by contracting R′; S is a general skeleton matrix in which a chain connected at the end represents a loop, and no loop represents S = S′; TS is an influence value skeleton matrix; WS is a loop labeling matrix; UP/DOWN hierarchy division represents how the reachable matrix R is subjected to hierarchical extraction of result priority and cause priority, respectively; D represents the influence degree; C represents the influenced degree; M represents the centrality degree; and R represents the cause degree.

#### Construction of a comprehensive influence matrix using the DEMATEL method

##### Establish the direct influence matrix O

Based on the 13 constraints identified by the literature analysis and interview survey, the Delphi method was used to organize a team of experts who were closely involved in the field and familiar with the NSSI of adolescents. The aim was to quantify and score the interactions between factors in a multidimensional index system.

The main subjects of the survey included five experienced parents with foundational knowledge of this field; seven adolescent teachers or school department managers; eight school psychological counseling teachers or social workers or activists in the field of adolescent services; and seven experts in the field of NSSI behavioral psychology. This formed an expert team comprising 27 people. A 0–4 scale (“0”: no impact, “1”: relatively weak impact, “2”: moderate impact, “3”: relatively strong impact and “4”: very strong impact) was used to collect all the expert scoring tables, from which the sum of each quantitative impact relationship in the 27 scoring tables was calculated.

After the analysis, it was determined that the factors influencing adolescent NSSI behavior were $S = \left\{ {S_{1} } \right.,\;S_{2} , \ldots ,\;\left. {S_{13} } \right\}$. The relationship between the factors and the degree of direct influence between *S*_*i*_ and *S*_*j*_ is represented by *O*_*ij*_. *O*_*ij*_ is the strength of the influence of Factor R on *j*. Finally, *O*(*O*_*ij*_)_13×13_ is the direct-influence matrix O.

##### Establish the comprehensive influence matrix T

The comprehensive influence matrix T was obtained by normalizing the direct influence matrix O, considering the direct and indirect influences between relevant factors, and then calculating the formula. Specifically, first, a direct influence matrix O was normalized through a row sum maximum method using formula (1) to obtain a standard influence matrix N. Then, the standard influence matrix was converted into a comprehensive influence matrix T using formula (2) by adopting a direct influence and indirect influence accumulation mode, where I is the identity matrix, and (*I* − *N*)^−1^ is the inverse of (*I* − *N*). The calculation formulas are as follows:

1 $$N = \left( {\frac{{O_{ij} }}{{Max\left( {\sqrt {a_{i}^{2} + b_{i}^{2} } } \right)}}} \right)_{13 \times 13}$$

2 $$T = \left( {t_{ij} } \right)_{13 \times 13} = N + N^{2} + N^{3} + \cdots + N^{k} = \sum\limits_{k = 1}^{\infty } {N^{k} \to T = N\left( {I - N} \right)^{ - 1} }$$

#### Construct a comprehensive influencing factor model through TISM

##### Establish an adjacency matrix

The adjacency matrix describes the direct relationship between every possible pair of factors in a system. In this study, the Delphi method was used to consult experts and scholars in the field. By studying the relationship between various factors, a 13 × 13 adjacency matrix was established^51^ to visually represent the relationships between these factors. If an influence relationship existed between the row and column factors in the system, it was recorded as “1”; if there was no influence, this was recorded as “0”. Subsequently, the concept of intercept λ was introduced. Using formulas (3)–(5), the adjacency matrix was obtained from $T\mathop{\longrightarrow}\limits^{\lambda }A$ to produce adjacency matrix A. Here, the intercept value was obtained from matrix T, where ${\overline{\text{x}}}$ is the mean value of matrix T, ${\overline{\text{x}}}$ = 0.0533500960597249, and $\sigma$ is the overall standard deviation. The calculation formulas are as follows:

3 $$\lambda = \overline{{\text{x}}} + \sigma$$

4 $$\sigma = \sqrt {\frac{{\sum\nolimits_{i = 1}^{{{\text{n}}^{2} }} {\left( {x_{i} - \overline{x}} \right)^{2} } }}{{n^{2} }}}$$

5 $$O_{ij} = \left\{ {\begin{array}{*{20}c} {1,\;\;e_{i} \to e_{j} ,\;\;t > \lambda = 0.0897844407\quad \quad 8272} \\ {0,\;\;e_{i} \to {\text{e}}_{j} ,\;\;t > \lambda = 0.0897844407\quad \quad 8272} \\ \end{array} } \right.$$

##### Establish the reachability matrix R

The reachability matrix R was obtained using the Boolean operation of the adjacency matrix and identity matrix. It describes the degree that can be reached between the nodes of the directed connection graph after a certain path length in the form of a matrix. First, a Boolean summation was performed on adjacency matrix A and identity matrix I using formula (6) to obtain multiplication matrix B. A matrix Boolean iterative operation was then performed on the multiplication matrix B using formula (7), and the subsequent formula was established to obtain the reachable matrix R. Here, B is the multiplication matrix and I is the identity matrix, that is, a square Boolean matrix with only one diagonal. The calculation formulas are as follows:

6 $$B = A + I$$

7 $$B^{{{\text{k}} - 1}} \ne B^{k} = B^{K + 1} = R$$

##### Establish a general skeleton matrix S

To simplify the structure of the entire system, the reachable matrix R was used for point reduction to obtain the point reduction matrix R′. The aim was to check the relationships between the strong link factors at each level of the reachable matrix, whereas the skip binary relationships between the factors with an adjacent binary relationship were deleted to obtain an edge reduction distance matrix S′. The essence of an edge-reduction operation is to delete repeated paths. Finally, elimination was performed for a binary relation that could be reached by itself in the contracted edge distance matrix S′; that is, all diagonal elements “1” of the contracted edge distance matrix were changed to “0” to obtain the general skeleton matrix S. When *S*_*i*_ and *S*_*j*_ satisfy the relation k_*i*_ = k_*j*_ = 1, then $S_{{\text{i}}}$ and $S_{j}$ are strongly connected. Formula (8) was used to calculate the contracted point matrix R′ to the contracted edge distance matrix S′ as follows:

8 $$S^{\prime} = R^{\prime} - \left( {R^{\prime} - I} \right)^{2} - I$$

##### Draw a scatter plot of centrality and causality

To understand the interrelation between, and the weight of influence of each variable in the system, the influence degree (D), influenced degree (C), centrality (M), and cause degree (R) of each element were calculated using formulas (9), (10), (11), and (12), respectively, while the analysis was undertaken by drawing a scatter diagram. The process is as follows.

First, the corresponding value in the T matrix was substituted into the S matrix at the place of “1” in the general skeleton matrix to obtain the TS matrix.

Then, we labeled the directed edge inside the loop as “1” to obtain the matrix WS with the influence value. The degree of influence, centrality, and cause degree of each element were further calculated according to the value t_*ij*_ of the comprehensive influence matrix T. t_*ij*_ represents the degree of direct influence plus the indirect influence of element *i* on element *j* as well as the degree of comprehensive influence of element *j* on element *i*, that is, the degree of comprehensive influence.

Finally, according to the influence value of each factor in the comprehensive influence matrix WS matrix, a structural scatter diagram of centrality and cause degree can be drawn. The following solution formulas were used to calculate the influence degree (D), influenced degree (C), centrality (M), and cause degree (R):

9 $$D_{i} = \sum\limits_{j = 1}^{n} {t_{ji} ,\quad (i = 1,\;2,\;3, \ldots ,\;n)}$$

10 $$C_{i} = \sum\limits_{j = 1}^{n} {t_{ji} \quad (i = 1,\;2,\;3, \ldots ,\;n)}$$

11 $$M_{i} = D_{i} + C_{i}$$

12 $$R_{i} = D_{i} - C_{i}$$

##### Perform hierarchical extraction

To better understand how the influencing factors affected the order of influence, cause-first and result-first extraction methods were adopted to establish the result-first up-type hierarchical map and the DOWN-type cause-first hierarchical map, respectively. The extraction rule for the up-type hierarchical graph is *T*(e_*i*_) = *R*(*e*_*i*_). As long as the reachable set is the same as the common set, relevant elements are extracted. The extracted elements were placed at the top each time, and the extracted elements were placed sequentially from top to bottom. The extraction rule of the DOWN-type hierarchical graph is *T*(*e*_*i*_) = *Q*(*e*_*i*_); that is, the extracted elements are placed below each time, and the extracted elements are placed in order from bottom to top.

The reachable matrix utilized a reachable set R, a look-ahead set Q, and a common set T, where T = R ∩ Q. Taking the adjacency matrix A as an example, the reachable set of e_*i*_ is denoted as *R*(*e*_*i*_), that is, all elements whose corresponding row values are “1”. The antecedent set of e_*i*_ is denoted by *Q*(*e*_*i*_); that is, the element corresponds to all the elements whose column value is “1”. The common set of *e*_*i*_ is denoted by *T*(*e*_*i*_) and *R*(*e*_*i*_) is denoted by *Q*(*e*_*i*_)^72^.

##### Draw a topology hierarchy

Based on the relationship between the elements and the extraction results of the antagonistic hierarchy, by integrating the influence values between the variables in the influence matrix, as well as by using the ranking results of centrality and causes, the game antagonistic directed topological hierarchy diagram with comprehensive influence values can be drawn. The reachable relationships between the influencing factors of adolescents' NSSI behavior are represented by directed line segments, and the two-way arrows indicate the formation of a loop, that is, a mutually reachable relationship. The lower layer indicates that the influencing factors are rooted, and the upper layer indicates that the influencing factors are direct.

### Ethics statement

This study was conducted in accordance with the Declaration of Helsinki and approved by the Human Research Ethics Committee of the Sultan Idris Education University Education (No. 2022-0465-01).

## Data Availability

Data used to support the findings of this study are available from the corresponding author upon request.
